# The complete genome sequence of *Staphylothermus marinus *reveals differences in sulfur metabolism among heterotrophic Crenarchaeota

**DOI:** 10.1186/1471-2164-10-145

**Published:** 2009-04-02

**Authors:** Iain J Anderson, Lakshmi Dharmarajan, Jason Rodriguez, Sean Hooper, Iris Porat, Luke E Ulrich, James G Elkins, Kostas Mavromatis, Hui Sun, Miriam Land, Alla Lapidus, Susan Lucas, Kerrie Barry, Harald Huber, Igor B Zhulin, William B Whitman, Biswarup Mukhopadhyay, Carl Woese, James Bristow, Nikos Kyrpides

**Affiliations:** 1 Genome Biology Program, Joint Genome Institute, Walnut Creek, USA; 2 Virginia Bioinformatics Institute, Virginia Polytechnic Institute and State University, Blacksburg, USA; 3 Department of Biochemistry, Virginia Polytechnic Institute and State University, Blacksburg, USA; 4 Department of Microbiology, University of Georgia, Athens, USA; 5 Joint Institute for Computational Sciences, University of Tennessee – Oak Ridge National Laboratory, Oak Ridge, USA; 6 Biosciences Division, Oak Ridge National Laboratory, Oak Ridge, USA; 7 Production Department, Joint Genome Institute, Walnut Creek, USA; 8 Project Management Department, Joint Genome Institute, Walnut Creek, USA; 9 Lehrstuhl für Mikrobiologie und Archaeenzentrum, Universität Regensburg, Regensburg, Germany; 10 Biological Sciences Department, Virginia Polytechnic Institute and State University, Blacksburg, USA; 11 Department of Microbiology, University of Illinois, Urbana USA; 12 Programs Department, Joint Genome Institute, Walnut Creek, USA; 13 Biological and Environmental Sciences Directorate, Oak Ridge National Laboratory, Oak Ridge, USA

## Abstract

**Background:**

*Staphylothermus marinus *is an anaerobic, sulfur-reducing peptide fermenter of the archaeal phylum Crenarchaeota. It is the third heterotrophic, obligate sulfur reducing crenarchaeote to be sequenced and provides an opportunity for comparative analysis of the three genomes.

**Results:**

The 1.57 Mbp genome of the hyperthermophilic crenarchaeote *Staphylothermus marinus *has been completely sequenced. The main energy generating pathways likely involve 2-oxoacid:ferredoxin oxidoreductases and ADP-forming acetyl-CoA synthases. *S. marinus *possesses several enzymes not present in other crenarchaeotes including a sodium ion-translocating decarboxylase likely to be involved in amino acid degradation. *S. marinus *lacks sulfur-reducing enzymes present in the other two sulfur-reducing crenarchaeotes that have been sequenced – *Thermofilum pendens *and *Hyperthermus butylicus*. Instead it has three operons similar to the *mbh *and *mbx *operons of *Pyrococcus furiosus*, which may play a role in sulfur reduction and/or hydrogen production. The two marine organisms, *S. marinus *and *H. butylicus*, possess more sodium-dependent transporters than *T. pendens *and use symporters for potassium uptake while *T. pendens *uses an ATP-dependent potassium transporter. *T. pendens *has adapted to a nutrient-rich environment while *H. butylicus *is adapted to a nutrient-poor environment, and *S. marinus *lies between these two extremes.

**Conclusion:**

The three heterotrophic sulfur-reducing crenarchaeotes have adapted to their habitats, terrestrial vs. marine, via their transporter content, and they have also adapted to environments with differing levels of nutrients. Despite the fact that they all use sulfur as an electron acceptor, they are likely to have different pathways for sulfur reduction.

## Background

Crenarchaeota is one of the two major phyla of the domain Archaea. Many crenarchaeotes are heterotrophic, anaerobic, sulfur-reducing hyperthermophiles, but the crenarchaeotes with completely sequenced genomes are primarily aerobes. Of the archaea with published genomes, only *Hyperthermus butylicus *and *Thermofilum pendens *are heterotrophic, obligate sulfur-reducing anaerobes [1,2]. More genomes are needed from anaerobic crenarchaeotes in order to determine if their phenotypic similarities are reflected in their genomes.

*Staphylothermus marinus *was isolated from a black smoker and from volcanically heated sediments [3]. It is a hyperthermophile, with a maximum growth temperature of 98°C. Its name reflects its proclivity to form clusters of up to 100 cells. At high concentrations of yeast extract it forms large cells up to 15 μm in diameter. It is a strict anaerobe and grows heterotrophically on complex media. H_2_S, CO_2_, acetate and isovalerate are metabolic products, suggesting a metabolism similar to that of the Thermococcales of the phylum Euryarchaeota. Dark granules observed within the cytoplasm may consist of glycogen. While *S. marinus *can survive in the absence of sulfur and produce hydrogen rather than H_2_S, it requires sulfur for growth [4]. An unusual cell surface protein named tetrabrachion has been characterized from *S. marinus *[5], and a 24-subunit phosphoenolpyruvate-utilizing enzyme with a unique structure has also been studied [6].

Here we report the complete genome of the anaerobic, sulfur-reducing archaeon *S. marinus *and a comparative analysis with other sulfur-reducing heterotrophic crenarchaeotes. While some features in *S. marinus *are similar to *H. butylicus *[7] and *T. pendens *[8], including peptide fermentation enzymes, there are also major differences, particularly in the electron transport machinery.

## Results

### General features

The genome of *S. marinus *F1 consists of a circular chromosome of 1.57 Mbp. There is one copy each of 5S, 16S, and 23S ribosomal RNA. About 59% of protein-coding genes begin with an AUG codon, 8% with GUG, and 33% with UUG. The low number of GUG start codons reflects the low GC content of this genome (35.7% GC). The ribosomal protein L12ae gene (Smar_1096) does not have a valid start codon, but this is likely to be an essential gene. Based on alignment with L12ae proteins from other archaea, it appears that the *S. marinus *gene begins with an ATC start codon. *S. marinus *has 12 regions of CRISPR repeats containing between 5 and 17 repeats. Twelve CRISPR-associated proteins are found in the vicinity of three of the repeats, between coordinates 323,400 and 345,500 (Smar_0308-Smar_0325), and one other CRISPR-associated protein is found at a different location not close to any repeats (Smar_1195).

The genome statistics for *S. marinus *and the two other sulfur-reducing crenarchaeotes are presented in Table 1. While the genome of *H. butylicus *is larger than that of *S. marinus*, they both have approximately the same number of genes due to the lower coding percentage of *H. butylicus*. *T. pendens *has a larger genome and a greater number of genes than the other two (discussed below). The GC content of *S. marinus *is much lower than the others, but this is not unusual for a hyperthermophile. It is in the same range as the GC content of the *Sulfolobus *genomes, while *Methanocaldococcus jannaschii *and *Nanoarchaeum equitans *have lower GC contents (31% and 32% respectively). *T. pendens *has a much higher percentage of genes in paralog clusters than the others, suggesting that gene duplication and divergence have been more prevalent in this genome. *S. marinus *has a smaller percentage of genes with signal peptides. In all three genomes the predicted exported proteins are primarily ABC transporter substrate-binding proteins and hypothetical proteins. *S. marinus *has approximately the same number of ABC transporters for uptake of nutrients as *H. butylicus*, but they both have fewer than *T. pendens*.

**Table 1:** Genome statistics.

	*S. marinus*	*H. butylicus*	*T. pendens*
Genome size (bp)	1,570,485	1,667,163	1,813,393

Coding region (bp)	1,399,012 (89.1%)	1,385,726 (83.1%)	1,651,626 (91.1%)

G+C content (bp)	561,080 (35.7%)	895,879 (53.7%)	1,045,351 (57.6%)

Total genes	1655	1668	1923

RNA genes	45 (2.7%)	52 (3.1%)	40 (2.1%)

Protein-coding genes	1610 (97.3%)	1616 (96.9%)	1883 (97.9%)

Genes with function prediction	974 (58.9%)	981 (58.8%)	1170 (60.8%)

Genes in ortholog clusters	1391 (84.0%)	1380 (82.7%)	1559 (81.1%)

Genes in paralog clusters	542 (32.7%)	488 (29.3%)	805 (41.9%)

Genes assigned to COGs	1109 (67.0%)	1114 (66.8%)	1264 (65.7%)

Genes assigned Pfam domains	1062 (64.2%)	1042 (62.5%)	1215 (63.2%)

Genes with signal peptides	37 (2.2%)	70 (4.2%)	134 (7.0%)

Genes with transmembrane helices	348 (21.0%)	294 (17.6%)	437 (22.7%)

Fusion genes	44 (2.7%)	32 (1.9%)	74 (3.8%)

*T. pendens *has about 270 more protein-coding genes than the other two, but only about 150 more genes with COG hits, suggesting that 120 of the additional genes in *T. pendens *are hypothetical proteins. We compared COG categories [9] between the three crenarchaeotes to determine what categories were more prevalent in *T. pendens *compared to the other two (Table 2). *T. pendens *has a higher number of genes in many categories, suggesting that the additional genes are spread out among a number of cellular processes. The three categories with the greatest additional genes in *T. pendens *are carbohydrate metabolism and transport, cell wall/membrane/envelope biogenesis, and function unknown. The greater number of carbohydrate-associated genes is mainly due to a larger number of transporters. *T. pendens *has more ABC transporters than the other two and a phosphotransferase (PTS) system transporter, as well as a higher number of transporters assigned to COG2814, arabinose efflux permease, which are transporters of the major facilitator superfamily. In addition, *T. pendens *has three sugar kinases of COG1070, while the other two have none. Thus *T. pendens *can probably take up and utilize a greater number of carbohydrates than the other two. The greater number of cell wall-associated genes in *T. pendens *is mainly due to a greater number of glycosyltransferases (COG0438) and nucleotide sugar metabolic enzymes. This suggests that *T. pendens *has a greater variety of sugars attached to lipids and/or proteins on the outside of the cell.

**Table 2:** Comparison of COG categories among the three sulfur-reducing crenarchaeotes.

	*S. marinus*	*H. butylicus*	*T. pendens*
Amino acid transport and metabolism	74	75	89

Carbohydrate transport and metabolism	72	40	108

Cell cycle control, cell division, chromosome partitioning	8	7	13

Cell motility	4	6	5

Cell wall/membrane/envelope biogenesis	23	24	47

Chromatin structure and dynamics	2	1	2

Coenzyme transport and metabolism	53	75	51

Cytoskeleton	0	0	1

Defense mechanisms	17	10	22

Energy production and conversion	92	109	119

Extracellular structures	0	0	0

Function unknown	116	113	132

General function prediction only	199	206	212

Inorganic ion transport and metabolism	85	57	82

Intracellular trafficking, secretion, and vesicular transport	12	15	15

Lipid transport and metabolism	15	20	20

Nuclear structure	0	0	0

Nucleotide transport and metabolism	39	43	42

Posttranslational modification, protein turnover, chaperones	53	56	63

RNA processing and modification	2	2	1

Replication, recombination and repair	71	61	78

Secondary metabolites biosynthesis, transport, and catabolism	5	9	3

Signal transduction mechanisms	18	24	15

Transcription	60	66	73

Translation	164	161	153

The *S. marinus *genome contains several protein families not found before in crenarchaeotes, and these are discussed below. *S. marinus *is the first crenarchaeote found to have an arginine decarboxylase belonging to COG1166 (Smar_0204), which includes the *speA *gene of *E. coli*. This protein family is also found in one euryarchaeote, *Methanosaeta thermophila*. Most euryarchaeota have a pyruvoyl-dependent arginine decarboxylase [10]. *T. pendens *and *Cenarchaeum symbiosum *also contain this type of enzyme. No arginine decarboxylase has been identified in other crenarchaeote genomes. Phylogenetic analysis of the *S. marinus *arginine decarboxylase (not shown) does not indicate a clear case of lateral gene transfer, and this enzyme was not identified during the search for laterally transferred genes (see below).

*S. marinus *contains a probable cell surface protein (Smar_0566) with 4 copies of the pfam03640 repeat, which has not been found in any other crenarchaeal genome. This repeat is present in two methanogens, Candidatus *Methanoregula boonei *and Candidatus *Methanosphaerula palustris*. It is also found in a wide variety of bacteria, but its function is unknown.

*S. marinus *is unique among crenarchaeotes in having a sodium ion-translocating decarboxylase for energy generation (Smar_1503-1504). It also has three putative operons containing subunits of multisubunit cation/proton antiporters, although these are likely to belong to large membrane-bound ion-translocating enzyme complexes rather than acting as cation antiporters (see below). *S. marinus *is the first crenarchaeote found to have a type I restriction-modification system (Smar_0761-0763).

**Figure 1:**
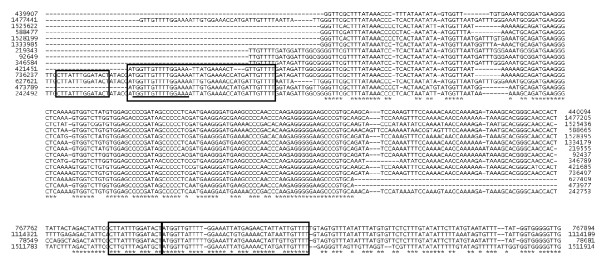
**Alignment of putative miniature inverted-repeat transposable elements (MITEs) from *S. marinus***. Start and end coordinates are given for each putative MITE. Below the MITEs are the upstream regions of four related transposases with start and end coordinates. The underlined sequences are the inverted repeats within the MITEs, and the boxed sequences are regions of similarity between the MITEs and the upstream regions of the transposases.

*S. marinus *has 5 putative transposable elements. Phylogenetic analysis shows that all of them belong to family IS607 (not shown). The characterized members of this IS family contain two ORFs. In *S. marinus *one of the elements contains two ORFs while the other four contain only one ORF. In the *S. marinus *element with two ORFs, the first (Smar_0846) is truncated relative to other members of the family, and is likely to be a pseudogene, while the second (Smar_0847) is intact. The four elements with one ORF share a high degree of similarity to each other (Smar_0083, Smar_0767, Smar_1150, Smar_1546), suggesting that they have been recently duplicated. In addition, there are 14 copies of a repeated sequence of approximately 260 nucleotides, although some of the repeats are truncated at one or both ends. These repeats are likely to be miniature inverted-repeat transposable elements (MITEs) as they are flanked by inverted repeats and have similarity to a region of DNA upstream of the group of four transposase ORFs (Figure 1). MITEs have previously been identified in some archaeal genomes [11]. Two ORFs are disrupted by MITEs, a protein with ABC transporter ATPase and acetyltransferase domains (Smar_0733) and a PIN domain protein (Smar_0327/0328). The presence of disrupted genes suggests that the MITEs have been active recently, although they do not appear to have had a major impact on the genome content.

Twenty-one probable laterally transferred genes were identified using the program SIGI-HMM [12]. One gene is by itself (Smar_0375), there are three pairs of genes (Smar_0568-0569, Smar_0846-0847, and Smar_1144-1145) and there is one cluster of 17 genes (Smar_1525-1541) in which 14 of the genes are predicted to be laterally transferred. Twelve of the laterally transferred genes are predicted to have come from other Crenarchaeota, six from Euryarchaeota, and the remaining three have unknown donors. Six of the 17 genes are likely to be pseudogenes, suggesting that they were transferred but then are degrading. From these findings we conclude that lateral transfer has not played a large role in shaping *S. marinus *gene content, and most if not all gene transfers have come from other archaea.

### Metabolism/transport

The presence of transporters for peptides and carbohydrates suggests that both types of compounds can serve as carbon and energy sources. *S. marinus *has four ABC transporters for carbohydrates (Smar_0088-0091, Smar_0108-0111, Smar_0299-0302, Smar_1146-1149) and two for peptides (Smar_0270-0274, Smar_0342-0346). It has a carbohydrate secondary transporter of the glycoside-pentoside-hexuronide (GPH) family (Smar_0710), and it is the first crenarchaeote found to have a peptide transporter of the oligopeptide transporter (OPT) family (Smar_1400). There are no ABC transporters for amino acids, but a probable amino acid transporter of the neurotransmitter:sodium symporter (NSS) family is present (Smar_0285). The presence of secondary transporters (GPH, OPT, and NSS), which have low affinity and high capacity, suggests that there are times when *S. marinus *is exposed to high levels of nutrients, and it can conserve energy by using secondary transporters instead of ATP-dependent transporters.

*S. marinus *has a glycolysis pathway similar to *Aeropyrum pernix*, with ATP-dependent glucokinase (Smar_1514) and phosphofructokinase (Smar_0007). Glycogen synthase (Smar_1393) and phosphorylase (Smar_0246) are present, suggesting that the dark granules observed in *S. marinus *cells are composed of glycogen. Similar to other crenarchaeotes and thermococci, *S. marinus *has pyruvate:ferredoxin oxidoreductase (Smar_1447-1450) and ADP-forming acetyl-CoA synthase (Smar_0449, Smar_1241-1242) for ATP synthesis from pyruvate. Three other 2-ketoacid:ferredoxin oxidoreductases are present (Smar_0291-292, Smar_0997-1000, Smar_1443-1444) that are probably involved in amino acid degradation.

**Figure 2:**
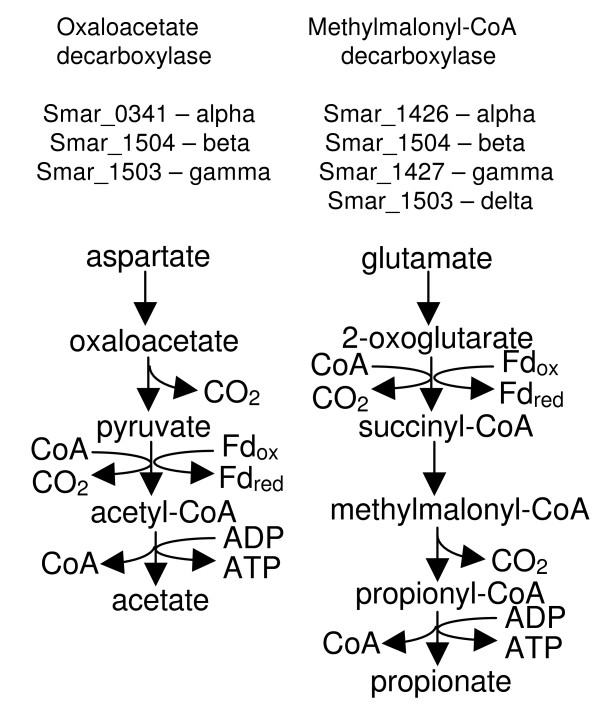
**Two possible functions of the sodium ion-translocating decarboxylase of *S. marinus *in their metabolic contexts**.

*S. marinus *is unique in Crenarchaeota in having a sodium-translocating decarboxylase. Smar_1504 and Smar_1503 encode the beta and gamma subunits (beta and delta in methylmalonyl-CoA decarboxylase). There are two possibilities for the activity of this decarboxylase (Figure 2). With Smar_1426 and Smar_1427 these genes could form a methylmalonyl-CoA decarboxylase. Smar_1426 and Smar_1504 are closely related to predicted methylmalonyl-CoA decarboxylase subunits of *Pyrococcus *species. This enzyme would be involved in catabolism of succinyl-CoA resulting from glutamate degradation via a 2-oxoacid:ferredoxin oxidoreductase (Figure 2). However, methylmalonyl-CoA mutase and epimerase were not found in the genome. The other possible function is oxaloacetate decarboxylase with Smar_0341 as the alpha subunit. This would be involved in catabolism of aspartate (Figure 2). However Smar_0341 is also related to pyruvate carboxylase B subunits of euryarchaeotes, and could interact with Smar_0140 to form this enzyme instead of or in addition to a sodium-transporting decarboxylase.

*S. marinus*, like the other heterotrophic crenarchaeotes *H. butylicus *and *T. pendens*, has lost almost all amino acid biosynthetic enzymes, although it has retained a few pathways for specific physiological reasons. For instance, glutamine is needed for its function as a nitrogen donor. Like the other heterotrophic crenarchaeotes, *S. marinus *can make pyrimidines but not purines. Enzymes for synthesis of several cofactors are present in *S. marinus*, in contrast to *T. pendens*, which lacks many cofactor synthesis pathways. *S. marinus *can likely synthesize riboflavin, pyridoxine, and coenzyme A, but it probably must acquire heme from the environment.

### Electron transport/sulfur reduction

*S. marinus *requires sulfur for growth and reduces it to sulfide [4], but it lacks homologs of proteins implicated in sulfur reduction in other organisms. It has no genes similar to sulfhydrogenases [13,14] and the recently discovered NADPH:sulfur oxidoreductase [15] from *P. furiosus*. It also lacks genes with similarity to the molybdoenzymes polysulfide reductase of *Wolinella succinogenes *[16], sulfur reductase of *Acidianus ambivalens *[17], sulfur reductase of *Aquifex aeolicus *[18], and thiosulfate/sulfur reductase of *Salmonella enterica *[19]. *S. marinus *has a gene (Smar_1055) with 56% similarity to sulfide dehydrogenase SudA subunit from *P. furiosus *[20], but this gene is shorter than the *P. furiosus *gene by 120 amino acids and the beta subunit is not present in *S. marinus*. Thus, this enzyme is unlikely to be present in *S. marinus*. However *S. marinus *has three putative operons similar in composition to the *mbh *and *mbx *operons of Thermococcales (Table 3). These multisubunit complexes are not found in any other sequenced crenarchaeote. The *mbh *operon from *P. furiosus *encodes a membrane-bound hydrogenase that oxidizes ferredoxin [21], while the *mbx *operon has a yet to be defined role in electron transfer. Its proposed function is the transfer of electrons from ferredoxin to NADPH coupled with proton translocation across the cell membrane [15]. A similar complex present only in *Pyrococcus abyssi *(PAB1395-1401) is adjacent to formate dehydrogenase subunits and has similarity to *E. coli *hydrogenases 3 and 4. Thus, it is likely to be a formate hydrogen lyase.

**Table 3:** Sulfur reduction enzymes and their presence in the three sulfur-reducing heterotrophic crenarchaeotes.

Enzyme	*S. marinus*	*T. pendens*	*H. butylicus*
Sulfur/polysulfide reductase (molybdoenzyme)	-	Tpen_1121-1123	Hbut_0371-0373

Sulfhydrogenase	-	-	-

Sulfide dehydrogenase	-	-	-

NADPH:sulfur oxidoreductase	-	Tpen_0143	Hbut_0802

*mbh*/*mbx*-related	Smar_0018-0030, Smar_0645-0657, Smar_1057-1071	-	-

**Figure 3:**
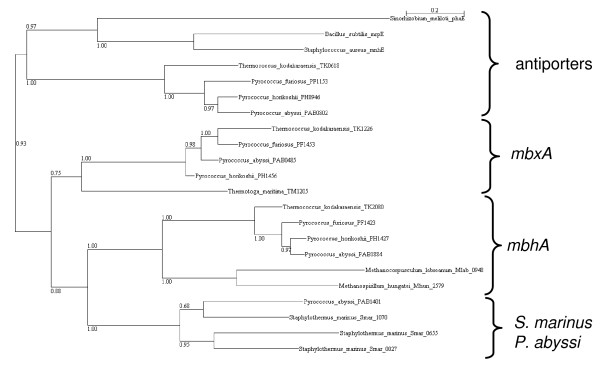
**Phylogenetic tree of proteins related to antiporter subunit *mnhE*/*mrpE*/*phaE***.

The *S. marinus mbh*/*mbx*-related complexes contain a set of proteins similar to components of multisubunit cation/proton antiporters and another set with similarity to NADH:ubiquinone oxidoreductase subunits (Table 4). The *S. marinus *antiporter-related subunits show high similarity to each other and to the corresponding subunits of the *P. abyssi *putative formate hydrogen lyase. *S. marinus *does not have an identifiable formate dehydrogenase, so these complexes likely have a different function in *S. marinus*. The *S. marinus *and *P. abyssi *proteins form a distinct cluster separate from *mbh *and *mbx *complexes and from the related cation/proton antiporters (Figure 3). In contrast, the NADH:ubiquinone oxidoreductase-related subunits in the *S. marinus *putative operons are not closely related to each other or to the corresponding *P. abyssi *formate hydrogen lyase proteins. These findings indicate that the antiporter-related subunits form a cassette that has been duplicated in *S. marinus *and combined with NADH:ubiquinone oxidoreductase-related subunits that are divergent in sequence.

**Table 4:** Subunit composition of multisubunit membrane-bound complexes from *Pyrococcus *species and *S. marinus*.

	*mbh*	*mbx*	PabFHL	Smar1	Smar2	Smar3
COG1863, MnhE	PF1423	PF1453	PAB1401	0027	0655	1070

COG2212, MnhF	PF1424	PF1452	PAB1398.1	0022	0650	1065

COG1320, MnhG	PF1425	PF1451	PAB1398	0023	0651	1066

COG1563	PF1426	PF1450	PAB1399.1	0024	0652	1067

COG2111, MnhB	PF1427 PF1428	PF1449	PAB1399	0025	0653	1068

COG1006, MnhC	PF1429	PF1448	PAB1400	0026	0654	1069

Pfam00361, MnhD/nuoLMN	PF1430	PF1447 PF1446	PAB1402 PAB1392 PAB1391	0028	0645	1057 1058 1071

mbhI-related	PF1431			0029		

Pfam01058, nuoB	PF1432	PF1444	PAB1396	0018	0646	1063

Pfam00329, nuoC	PF1433	PF1443	PAB1394	0019	0647	1061

Pfam00346, nuoD	PF1434	PF1442	PAB1394	0020	0648	1061

Pfam00146, nuoH	PF1435	PF1445	PAB1393	0030	0657	1060

COG1143, FHL6/nuoI	PF1436	PF1441	PAB1395	0021 (pseudo)	0649	1062

*S. marinus *produces hydrogen when sulfur is limiting [4]. Two of the multisubunit complexes are potentially involved in hydrogen production. One set of *S. marinus *proteins (Smar_1060-Smar_1063) clusters strongly with *E. coli *hydrogenases 3 and 4 in phylogenetic trees, and may form a membrane-bound hydrogenase. Smar_0018 and Smar_0020 have similarity (61% and 39%, respectively) to subunits of *Methanosarcina mazei *ech hydrogenase subunits, and hydrogenase accessory proteins are found in their vicinity (Smar_0012-0013, Smar_0015). It is likely that at least one of these clusters is involved in hydrogen production.

The other complexes may be involved in sulfur reduction either directly or indirectly. One of the clusters (Smar_1057-1071) is close on the chromosome to a pyridine nucleotide-disulfide oxidoreductase (Smar_1055). It is possible that this cluster is involved in sulfur respiration, where Smar_1055 acts as a NAD(P)H-dependent polysulfide reductase and the other ORFs are involved in the generation of NAD(P)H through a membrane-based electron transport system that oxidizes reduced ferredoxin and translocates protons across the membrane. The system would allow energy generation from an overall sulfur-dependent oxidation of peptides and amino acids and it would be similar to the *mbx*-NAD(P)H elemental sulfur oxidoreductase (NSR) system that has been described for *P. furiosus *[15].

### Comparison of the three sulfur-reducing crenarchaeotes

**Figure 4:**
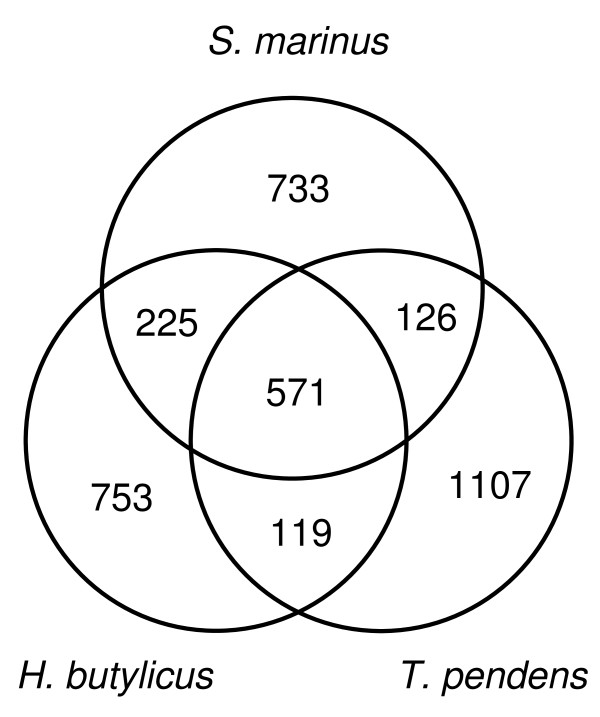
**Venn diagram showing genes shared between *S. marinus*, *H. butylicus*, and *T. pendens***.

Spectral clustering was used to create protein clusters from the three anaerobic sulfur-reducing heterotrophs, and the clusters shared by all three or by pairs of the three were derived (Figure 4) and [see Additional file 1]. The three organisms share 571 core clusters, somewhat more than the conserved crenarchaeal core of 336 determined by Makarova et al. [22]. Among the clusters conserved among the three but not found in all Crenarchaeota are the subunits of ABC transporters for sugars, peptides, and amino acids, which are required for their heterotrophic lifestyle. Also falling into this group are the ferrous iron transporter proteins FeoA and FeoB and the anaerobic form of ribonucleotide reductase, proteins which reflect their anaerobicity. *S. marinus *and *H. butylicus *have almost twice as many shared clusters (225) as either one has with *T. pendens *(119 or 126). This is due to their closer phylogenetic relationship. *S. marinus *and *H. butylicus *both belong to the order Desulfurococcales while *T. pendens *belongs to the order Thermoproteales.

The major difference in habitat between these three organisms is that *S. marinus *and *H. butylicus *were isolated from marine environments [1,3] while *T. pendens *was isolated from a terrestrial solfatara [2]. Marine environments have relatively high concentrations of sodium and potassium compared to terrestrial springs, and this influences the complement of transporters encoded by the three genomes. For example, *S. marinus *and *H. butylicus *use the Trk type of potassium transporter (COG0168), which is a proton or sodium symporter, while *T. pendens *uses the more energy-intensive ATP-dependent kdp-type potassium transporter (COG2060, COG2216, COG2156). Also, *S. marinus *and *H. butylicus *have a greater number and variety of sodium symporters than *T. pendens*. They both have sodium-dependent multidrug efflux pumps of the MATE family (COG0534) and amino acid transporters of the neurotransmitter:sodium symporter family (pfam00209), while only *S. marinus *has a transporter of the sodium:solute symporter family (pfam00474).

Both *T. pendens *and *H. butylicus *have formate dehydrogenases while *S. marinus *lacks this enzyme. Formate can be used as an electron donor with sulfur as electron acceptor to generate energy. *S. marinus *also lacks the FdhE protein, which is involved in formate dehydrogenase formation, while the other two have it.

There are also differences in the ability to utilize carbohydrates among the three organisms. As discussed above, *T. pendens *has a greater number of carbohydrate transporters than the other two. According to the CAZy database [23], *H. butylicus *has no glycosyl hydrolases, while *S. marinus *has ten and *T. pendens *has fifteen. Also *H. butylicus *apparently does not store glycogen as it lacks glycogen synthase and phosphorylase, but the other two have these. *H. butylicus *also lacks enzymes for utilization of galactose and N-acetylglucosamine. Surprisingly while *S. marinus *and *T. pendens *have probable glucokinases related to the characterized *Aeropyrum pernix *enzyme [24], *H. butylicus *has a protein related to the broad-specificity hexokinase from *Sulfolobus tokodaii *[25]. This suggests that, while it may not be able to break down polysaccharides, it may be able to utilize simple sugars.

There are similarities and differences among the three genomes in the genes involved in biosynthesis. Many of the genes shared by *S. marinus *and *H. butylicus *but missing from *T. pendens *are involved in cofactor metabolism. *T. pendens *appears to be unable to make riboflavin, coenzyme A, pyridoxine, and possibly other cofactors, and it has transporters for biotin and riboflavin that are not found in the other two. Among the three organisms only *H. butylicus *has a heme biosynthesis pathway. On the other hand, all three organisms are unable to make most amino acids and purines, although they do have the pyrimidine biosynthetic pathway. *S. marinus *and *H. butylicus *have ABC transporters of the basic membrane protein family (pfam02608) that probably transport nucleosides [26], but *T. pendens *lacks this type of transporter. In fact *T. pendens *does not have any identifiable nucleoside or nucleobase transporters, so it likely has undiscovered families to transport these compounds.

There are other differences between these three organisms that do not directly reflect the habitats they live in. *H. butylicus *is surprisingly lacking some enzymes of central metabolism. It has no identifiable fructose-bisphosphate aldolase and no phosphoenolpyruvate synthase or pyruvate phosphate dikinase. Since fructose-bisphosphate aldolase is essential for hexose and pentose synthesis, it likely has a new version of this enzyme. *H. butylicus *also does not have an asparaginyl-tRNA synthetase; however, it is the only one of the three to have an Asp-tRNA(Asn)/Glu-tRNA(Gln) amidotransferase, but the A subunit of this enzyme (Hbut_0594) has a frameshift. Since this appears to be an essential enzyme for *H. butylicus*, the gene may still be functional.

## Discussion

The crenarchaeotes *H. butylicus*, *S. marinus*, and *T. pendens *are similar phenotypically in that they all degrade peptides and/or carbohydrates using 2-oxoacid:ferredoxin oxidoreductases, they are anaerobes, and they are dependent on sulfur reduction to dispose of electrons. They all have genomes in the size range of 1.6–1.8 Mbp. Having these three genome sequences allows comparative studies to determine whether their phenotypic similarity is reflected in their genome sequences.

While the central metabolic pathways for generation of ATP from peptides appear to be similar between the three, there are also differences. For instance there are different sets of transporters used by the marine organisms versus the terrestrial one. However the biggest differences between the three relate to the availability of nutrients. On one extreme is *H. butylicus*, which has no glycosidases and is capable of synthesizing most if not all cofactors it needs. This organism appears to be more specialized than the other two in that it is restricted to the use of peptides and amino acids as energy sources, although formate can also be utilized. On the other extreme is *T. pendens *which has many glycosidases and relies on its environment for most cofactors, thus it is used to being in a nutrient-rich environment. Probably a terrestrial solfatara environment allows nutrients to be concentrated as compared to marine environments in which nutrients may be quickly dispersed. *S. marinus *falls in the middle ground as it has several glycosidases like *T. pendens *but it encodes most cofactor biosynthesis pathways like *H. butylicus*. Its use of secondary transporters and ABC transporters suggests that at least at some times it is exposed to high levels of nutrients. It is adapted to an environment that contains carbohydrates as well as proteinaceous substrates, but in which cofactors are not present at high levels.

Characterized membrane-bound sulfur and polysulfide reductases have three subunits [16-19]. The A and B subunits are related in sequence, but the C subunits belong to different protein families. *T. pendens *and *H. butylicus *have putative three-subunit sulfur reductases in which all subunits are adjacent on the chromosome. These complexes have the standard A and B subunits, but they differ in their C subunits. The *T. pendens *C subunit belongs to the same family as the *W. succinogenes psrC *subunit, while the *H. butylicus *C subunit is related to *sreC *of *Acidianus ambivalens*. *S. marinus *lacks this type of sulfur or polysulfide reductase, and it is the only crenarchaeote other than *C. symbiosum *to lack this family of molybdopterin oxidoreductases (COG0243).

*T. pendens *and *H. butylicus *also have putative NADPH:sulfur oxidoreductases similar to the *P. furiosus *enzyme [15], which is also absent in *S. marinus*. However, *S. marinus *has three *mbh*/*mbx*-related multisubunit complexes, which are not found in the other two genomes. The overall picture of sulfur reduction shows that *T. pendens *and *H. butylicus *may use similar pathways, while *S. marinus *uses different ones. This is in contrast to the phylogenetic positions of these organisms: *S. marinus *and *H. butylicus *belong to the order Desulfurococcales, while *T. pendens *belongs to the order Thermoproteales. The molybdoenzymes are widespread within Crenarchaeota, missing only in *S. marinus*, and may represent the ancestral path for sulfur reduction in Crenarchaeota. This analysis, however, rests on comparison to sulfur reduction enzymes characterized in other organisms, and new sulfur reduction pathways may be identified in the future.

## Conclusion

The three heterotrophic sulfur-reducing crenarchaeotes have adapted to their habitats, terrestrial vs. marine, via their transporter content, and they have also adapted to environments with differing levels of nutrients, with *T. pendens *being adapted to a nutrient-rich environment and *H. butylicus *adapted to an environment in which only peptides are present. *S. marinus *appears to have different electron transport pathways compared to the phenotypically similar organisms *T. pendens *and *H. butylicus*, showing that this phenotype is not encoded by the same genotype in these organisms.

## Methods

*S. marinus *strain F1 is available from the Deutsche Sammlung von Mikroorganismen und Zellkulturen (DSMZ) as DSM 3639^T ^and from the American Type Culture Collection (ATCC) as ATCC 43588. *T. pendens *Hrk5 is available from DSMZ as DSM 2475, and *H. butylicus *is available from DSMZ as DSM 5456. *S. marinus *F1 cells were grown in a 300 liter fermenter at 85°C in SME medium with 0.1% yeast extract, 0.1% peptone, and 0.7% elemental sulfur under a 200 kPa N_2 _atmosphere. Cells grew to a density of 3 × 10^8 ^cells/ml in 3 days. Cell pellets were stored at -85°C. DNA was extracted based on the method of Zhou et al. [27]. One gram of cells was dissolved in 4.5 ml extraction buffer (100 mM Tris, pH 8.0, 100 mM EDTA, 100 mM sodium phosphate, and 1.5 M NaCl). After 200 micrograms of proteinase K were added, cells were incubated for 30 minutes at 37°C. A solution of 0.5 ml 20% SDS was added, and then the mixture was incubated at 65°C for 2 hours. Proteins were removed by extraction with 5 ml phenol. The sample was centrifuged for 30 minutes at 19000 rpm in a Sorvall SS34 rotor at 10°C, and the upper phase was discarded. The sample was then extracted twice with chloroform and isoamyl alcohol (24:1) to remove phenol. DNA was precipitated with 3 ml isopropanol at room temperature overnight. The sample was then centrifuged for 30 minutes. The pellet was washed with 5 ml 70% ethanol and recentrifuged. The pellet was dried and then dissolved in 1 ml LiChrosolv (Merck, Darmstadt, Germany). RNA was removed by addition of 20 μg DNAse-free RNAse and incubation for 4 hours at 37°C.

The genome of *S. marinus *was sequenced at the Joint Genome Institute (JGI) using a combination of 3 kb, 8–10 kb and 40 kb (fosmid) DNA libraries. For all three libraries, shearing is followed by blunt end repair; then the DNA is isolated on an agarose gel and the appropriate section of the gel is cut out. For fosmid libraries, DNA is separated on a pulsed-field gel. DNA is extracted from the gel and then cloned into the pUC18 vector for 3 kb libraries, the pMCL200 vector for 8–10 kb libraries, or the pCC1FOS vector for 40 kb fosmid libraries. Sequencing is carried out from both ends of the inserts using BigDye Terminators and ABI3730XL DNA sequencers. More detailed information about library construction and sequencing, including protocols and reagents, is available at . Draft assemblies were based on 23766 total reads. All three libraries provided 13.3× coverage of the genome. The Phred/Phrap/Consed software package  was used for sequence assembly and quality assessment [28-30]. All mis-assemblies were corrected and all gaps between contigs were closed by custom primer walk using subclones or PCR products as templates. A total of 657 additional reactions were necessary to close gaps and to raise the quality of the finished sequence. The Phred quality score for this genome is Q50, which corresponds to one miscalled base per 100,000 bases. The genome sequence of *S. marinus *can be accessed in GenBank [GenBank: CP000575]. The Genomes On Line Database (GOLD) accession number is . Genes were identified using a combination of Critica [31] and Glimmer [32] followed by a round of manual curation.

Analysis of the *S. marinus *genome was carried out with the Integrated Microbial Genomes (IMG) system [33]. Proteins unique to *S. marinus *or missing from *S. marinus *but present in other crenarchaeotes were identified with the phylogenetic profiler in IMG. Transposable elements were identified by BLAST against the ISFinder database [34]. CRISPR repeats were identified with the CRISPR Recognition Tool [35].

Laterally transferred genes were identified with SIGI-HMM [12]. DNA and protein alignments were generated with CLUSTAL W [36]. The phylogenetic tree was generated with MrBayes 3.1.2 [37] with 1,000,000 generations sampled every 100 generations. The first 250,000 generations were discarded as burn-in. The tree was viewed and manipulated with njplot [38].

To generate clusters for comparative genomics, we retrieved all amino acid sequences for *S. marinus*, *H. butylicus*, and *T. pendens *along with their blastp [39] (e-value < 10-6) similarity scores, from the Integrated Microbial Genomes database [33]. Thereafter, we divided the resulting network of protein similarities into distinct similarity matrices. Each matrix (cluster of proteins) was then successively partitioned into two child clusters using a spectral clustering procedure [40,41]. This procedure is analogous to a random walk of a particle moving over the proteins of the network. At each transition, the particle moves to an adjacent protein with probabilities corresponding to the similarity between proteins. The amount of time the particle spends in a given sub-network will determine whether this is indeed a cluster of its own or not. The magnitude of the second eigenvalue of the similarity matrix for a network will determine how fast the particle approaches its stationary distribution [42]. Here, we chose to partition the network if the second eigenvalue was 0.8 or more. This approach resulted in 1041 clusters of a total of 2653 proteins with homologs within two or more of the organisms.

## Authors' contributions

HH isolated the DNA, SL and AC carried out the sequencing and assembly, HS and AL finished the genome, ML carried out gene calling and annotation. IA, LD, JR, IP, LEU, SH, JGE, KM, and BM contributed to the analysis. IA compiled the manuscript. HH, IBZ, WBW, BM, CW, JB, and NK supervised aspects of the project and critically reviewed the manuscript. All authors approved the final manuscript.

## Supplementary Material

Additional file 1 **Clusters shared between two or three of the genomes *S. marinus*, *H. butylicus*, and *T. pendens***. List of cluster IDs with the protein family they belong to, followed by a list of locus tags for proteins belonging to the cluster. Click here for file
